# A Power-Efficient Multiband Planar USB Dongle Antenna for Wireless Sensor Networks

**DOI:** 10.3390/s19112568

**Published:** 2019-06-05

**Authors:** Wei-Yuan Chiang, Chia-Hao Ku, Chiung-An Chen, Le-Yu Wang, Patricia Angela R. Abu, Pei-Zong Rao, Chih-Kai Liu, Chao-Hsiang Liao, Shih-Lun Chen

**Affiliations:** 1 Department of Electrical Engineering, Ming Chi University of Technology, New Taipei City 24301, Taiwan; jameschiang@mail.mcut.edu.tw (W.-Y.C.); kuchiahao@mail.mcut.edu.tw (C.-H.K.); M011F8020@mail2.mcut.edu.tw (L.-Y.W.); 2 Department of Information Systems and Computer Science, Ateneo de Manila University, Quezon City 1108, Philippines; pabu@ateneo.edu; 3 Shenzhen Jaguar Wave Technology Co. Ltd., Shenzhen 518103, China; peizong.rao@jaguarwave.com (P.-Z.R.); chihkai.liu@jaguarwave.com (C.-K.L.); 4 Wireless Laboratory, SGS Taiwan Ltd., New Taipei City 24803, Taiwan; peter.liao@sgs.com; 5 Department of Electronic Engineering, Chung Yuan Christian University, Taoyuan City 32023, Taiwan

**Keywords:** multiband planar, USB dongle antenna, wireless sensor networks

## Abstract

Wireless Sensor Networks (WSNs) had been applied in Internet of Things (IoT) and in Industry 4.0. Since a WSN system contains multiple wireless sensor nodes, it is necessary to develop a low-power and multiband wireless communication system that satisfies the specifications of the Federal Communications Commission (FCC) and the Certification European (CE). In a WSN system, many devices are of very small size and can be slipped into a Universal Serial Bus (USB), which is capable of connecting to wireless systems and networks, as well as transferring data. These devices are widely known as USB dongles. This paper develops a planar USB dongle antenna for three frequency bands, namely 2.30–2.69 GHz, 3.40–3.70 GHz, and 5.15–5.85 GHz. This study proposes a novel antenna design that uses four loops to develop the multiband USB dongle. The first and second loops construct the low and intermediate frequency ranges. The third loop resonates the high frequency property, while the fourth loop is used to enhance the bandwidth. The performance and power consumption of the proposed multiband planar USB dongle antenna were significantly improved compared to existing multiband designs.

## 1. Introduction

The increasing use of portable devices with low-power integrated circuits and wireless communication has paved its way to a new generation of wireless sensor area networks. Simultaneously there is a trend towards the miniaturization of devices. In wild band use, this would refer to wireless networks that consist of several small body sensor units with a central control unit. Therefore, Chen et al. [1] used a micro-control unit design that covers all of the wireless body sensor nodes and manages a low power usage. Moreover, Chen et al. [2] proposed a reconfigurable filter to adjust the different biomedical signal inputs in order to optimize system performance.

Since the growth of use of wireless communication systems, processing requires a higher frequency and a wider transfer band. The data type of Wireless Body Area Networks (WBANs) is no longer limited to data in digits. With this, the new wireless communication specification and its power consumption increased drastically. Chen et al. [3] proposed an efficient lossless compression design. By efficiently compressing the data, the transfer speed and the power consumption were improved. Thus, the WBAN field could integrate a real-time health monitoring system that can continuously update the sensor records through the Internet and has also become an important interdisciplinary domain.

The continuous development in wireless communications improves the efficiency of an antenna by designing a smaller one that is more useful in terms of radiative properties. This led the way to research on multiband antenna. The Multiple-Input and Multiple-Output (MIMO) system is one typical case of the use of multiband antenna. Printed antennas have demonstrated that it can be useful, not only as a communicating element, but also as a sensor or energy collector. With this, the multiband antenna design has become an extensive subject. As a result, the use of a multiband antenna system on mobile devices is a common trend [4].

In recent years, the more compact and more powerful devices are the major design trends. Among those is the multiband antennas for Universal Serial Bus (USB) dongles that have been of particular interest due to the ease of carrying them as well as their plug and play function. The common USB dongle antenna design adopted the C-type line, combined with open or short stubs [4,5,6]. The stubs can also generate the low band [7,8,9,10]. In [11,12,13,14,15,16,17], the multi-path coupling antenna design was described. The main antenna and stubs are used to generate both the low band and middle band. The high band needs two coupling stubs in order to generate a wider bandwidth.

The reduction of the volume of the antenna is under the constraint of its fundamental physical limits in terms of trade-off between radiation performances and impedance bandwidth. Limitations in terms of bandwidth and efficiency suggest an analysis with respect to fundamental limits. The Chu’s model uses spherical modes to estimate the minimum stored energy around the antenna [18]. Equation (1) shows the modified formula of the Q-factor and the antenna size [18,19]:

(1) $$Q=\frac{2\omega \cdot \max\left\{W_{m},W_{e}\right\}}{P_{r}+P_{\Omega }}\approx \eta \left(\frac{1}{{\left(ka\right)}^{3}}+\frac{1}{ka}\right)$$

where *W_e_* is the stored electric energy, *W_m_* is the stored magnetic energy, *P_r_* is the radiated power, *P*_Ω_ is the power dissipated from ohmic losses, *η* is the radiation efficiency, *a* is the minimum radius of the sphere enclosing the antenna, and *k* is the wave number (*k* = 2π/λ). It is very difficult to have a wide bandwidth (low Q-factor) and at the same time have a good efficiency for miniature antennas. Thus, the miniaturization of antennas causes them to suffer from both limited efficiency and low bandwidth. Miniaturization of devices leads to the reduction of antennas, thus becoming an important challenge. Otherwise, the radiation efficiency decreases when the size of the antenna is reduced. For example, the meander line antenna is a small antenna that has a simple structure and can support higher bandwidth, but these antennas have low radiation efficiency [20].

**Figure 1:**
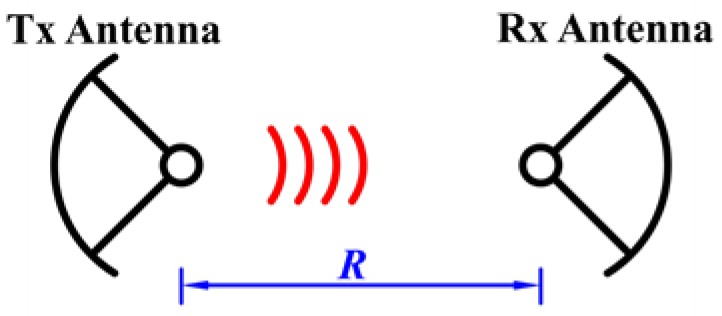
The schematic diagram of the communication system.

The important parameters of the antenna design are its operation frequency bands, radiation efficiency, and peak gain. The first work estimated the radiation efficiency. Figure 1 shows a schematic diagram of the communication system. The received power (*P_rx_*) is estimated using the Friis transmission equation shown in Equation (2) where *P_tx_* is the transmission power, *G_tx_* is the transmission antenna gain, *G_rx_* is the received antenna gain, *λ* is the free space wavelength, and *R* is transmission distance [21]. *G_rx_* is the 3D average gains, defined in Equation (3). Using an omnidirectional antenna to transmit the signal has a *G_tx_* of 0 dBi. The receiver minimum input level sensitivity is −61 dBm, which meets the specification of IEEE (Institute of Electrical and Electronics Engineers) [22]. If the transmission port is an ideal isotropic antenna, the *G_rx_* gain must be larger than −17 dBi when the frequency is 2.45 GHz. In addition, the transition range is 5 m with a transmission power of 10 dBm. The minimum radiation efficiency must be larger than 2%, and it can be calculated using Equation (3). However, the minimum radiation efficiency must be larger than 4% when the frequency is 2.45 GHz. On the other hand, the minimum radiation efficiency must be larger than 10% when the frequency is 5.5 GHz.

(2) $$P_{rx}=\frac{P_{tx}G_{tx}G_{rx}\lambda^{2}}{{\left(4\pi R\right)}^{2}}$$

(3) 3D average gain=10 log(radiation efficiency)

The next main parameter is the peak gain. The ideal USB dongle antenna is an omnidirectional antenna. In this study, the design goal is to have a peak gain within 0–4 dBi. This antenna is operated on LTE (Long Term Evolution), which covers 2.30–2.69 GHz, 3.40–3.70 GHz, and 5.15–5.85 GHz bands, 5G system (5th generation system) that covers 3.40–3.70 GHz, and the LTE-U (LTE-Unlicensed) band that covers 2.40–2.44 GHz and 5.15–5.85 GHz [23,24].

In this study, a planar antenna is designed to satisfy the conditions of low cost, miniaturized, and multiband antennas, taking into consideration the size and its production. It is challenging to design a multiband antenna as the structure gets smaller and smaller. The goal of this study is to design a triple band antenna that has a 10% radiation efficiency at three operating frequency bands with a peak gain within 0–4 dBi for each band.

The planar USB dongle is proposed in this work. The planar USB dongle antenna is a low power device that satisfies the condition of a low power system design. The proposed design combines one main feed antenna and three stubs in order to generate triple bands within a 10 mm × 10 mm area. The main feed antenna is responsible for generating the low band and middle band. One stub is incorporated to couple with the main antenna for the 2.30–2.69 GHz and 3.40–3.70 GHz bands. The second stub is incorporated to generate the high band. Lastly, the third stub is incorporated to increase the high band bandwidth in order to cover the 5.15–5.85 GHz band. Section 2 presents the antenna design and optimization. The performance comparison of the proposed antenna to previous works is presented and discussed in Section 3.

## 2. Antenna Design and Optimization

**Figure 2:**
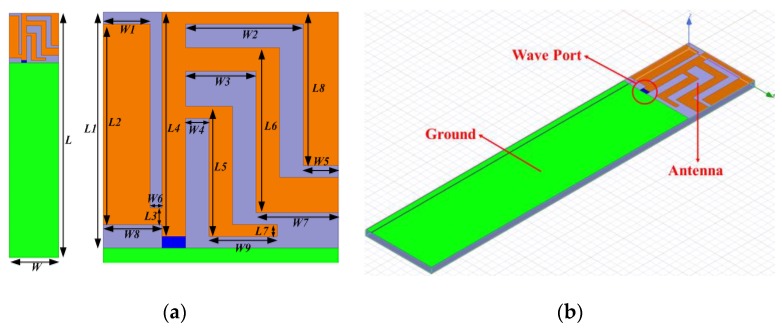
(**a**) The structure and (**b**) HFSS simulation model of the planar Universal Serial Bus (USB) dongle antenna.

The structure of the planar USB dongle antenna proposed in this study is illustrated in Figure 2a. The size of the main structure is 10 × 10 mm^2^, while the volume of the planar USB dongle antenna assembly is 10 × 50 × 0.8 mm^3^. The detailed dimensions of the planar USB dongle antenna are listed in Table 1.

The proposed antenna is designed on an FR4 substrate. Its measured relative permittivity and loss tangent are 4.4 and 0.02, respectively. The antenna is operated on LTE, which covers 2.30–2.69 GHz, 3.40–3.70 GHz, and 5.15–5.85 GHz bands, and the 5G system (Sub 6 GHz spectrum), which covers 3.40–3.60 GHz bands. Otherwise, the LTE-U band of the 5G system that covers 2.40–2.44 GHz and 5.15–5.85 GHz is included in the design goal of this antenna.

Figure 2b illustrates the simulation model that is constructed using the high frequency structure simulator, ANSYS HFSS EM software. The design targets three bands: (1) The lower band that covers 2.30–2.69 GHz, (2) the middle band that covers 3.40–3.70 GHz, and (3) the upper band that covers 5.15–5.85 GHz.

**Figure 3:**
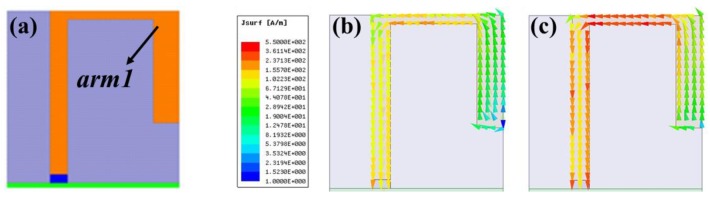
(**a**) The structure of *arm1* and the surface current distribution of *arm1* at (**b**) 2.45 GHz and (**c**) 3.68 GHz.

**Figure 4:**
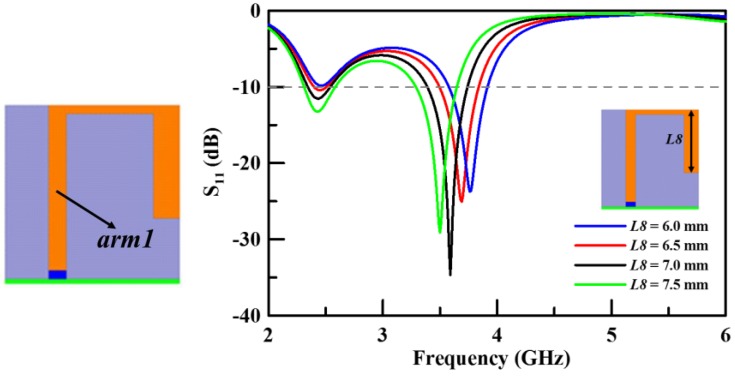
The simulated return loss with various lengths of L8 (Step 1).

The proposed multiband planar USB dongle antenna is constructed using two main antennas (*arm1* and *arm2*) and two stubs (*stub1* and *stub2*). The design and optimization of the proposed antenna involves four steps (Step 1–4). The first step (Step 1) uses an arm to resonate the modes in the lower and middle bands. This arm is called *arm1* and is shown in Figure 3a. The main antenna is specifically labelled *arm1* with a length of 21 mm. Its lower band resonates, which satisfies the resonated condition of a quarter wavelength. Figure 3b shows the surface current distribution on *arm1* at 2.45 GHz. On the other hand, Figure 3c illustrates the middle band, which satisfies the resonated condition of a half wavelength. This is the second resonant mode of *arm1*. Figure 4 shows the simulation results of the return loss with various *L8* parameter values for *arm1*. In Step 1, the parameter *L8* decides the total length of *arm1* and optimizes the frequency range of the lower and middle bands. The operation frequency range is closest to the design goal when *L8* is 6.5 mm. The impedance match is poor in the lower band, which leads to a small operation bandwidth. The succeeding steps of the design can improve on this by adding a stub.

**Figure 5:**
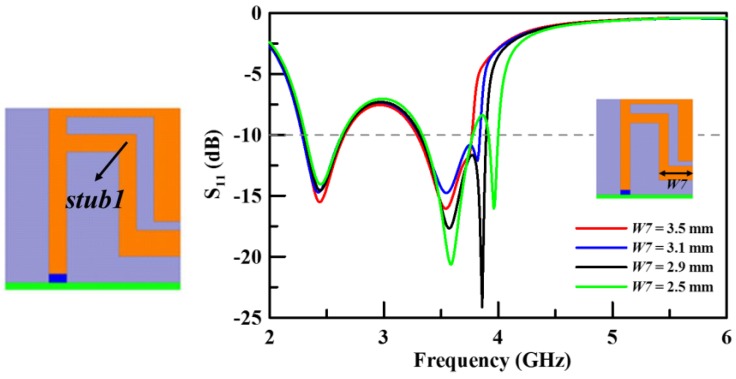
The simulated return loss with various lengths of *W7* (Step 2).

The second step (Step 2) of the design is to add an inductive stub, labelled *stub1* as shown in Figure 5. This can neutralize the capacitive circuit in order to enhance the bandwidth. The enhancement of the bandwidth of the lower band is clearly illustrated in Figure 5, which shows the simulation results of the return loss. The parameter *W7* is an important parameter for *stub1*. *Stub1* clearly enhances the bandwidth of the lower band. Otherwise, that can impact the characteristic of the middle band. Figure 5 illustrates the simulation results of the return loss with various *W7* parameter values for *stub1*. There are two modes on the spectrum of the middle band when *W7* is at 3.1 mm, 2.9 mm, and 2.5 mm, reducing the bandwidth of the middle band of the final structure. The bandwidth of the middle band is not at its widest when *W7* is at 3.5 mm, but it is at its best size for this design.

**Figure 6:**
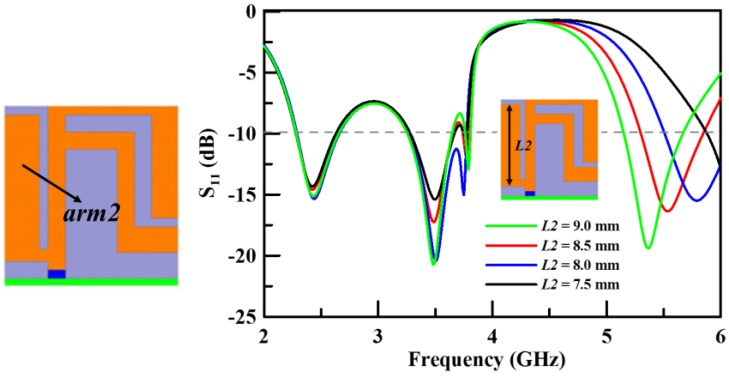
The simulated return loss with various lengths of *L2* (Step 3).

At the third step (Step 3), another main arm (*arm2*) is added in the circuit in order to resonate the upper band. Figure 6 shows the structure of *arm2* and illustrates that *arm2* resonates the upper band. This design satisfies the resonated condition of a quarter wavelength. The parameter *L2* is an important variable factor of *arm2* to decide the frequency range of the upper band. Figure 6 illustrates the simulation results of the return loss with various *L2* parameter values for *arm2*. The operation frequency range is closest to the design goal when *L2* is 8.5 mm.

**Figure 7:**
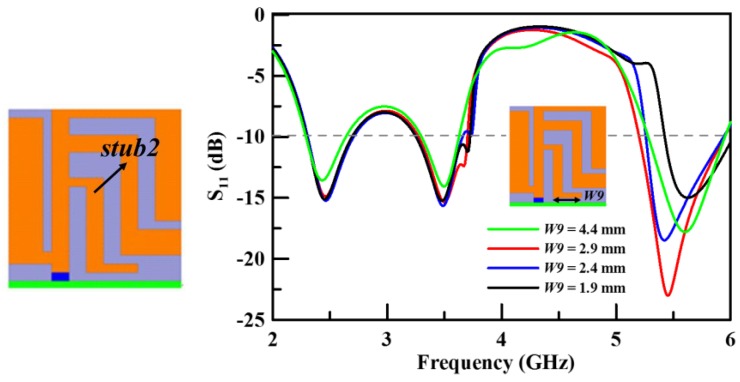
The simulated return loss with various lengths of *W9* (Step 4).

The fourth and last step (Step 4) incorporates an inductive stub (*stub2*) that can neutralize the capacitive circuit to enhance the bandwidth of the upper band. The main function of *stub2* is to tune the upper band impedance for the proposed antenna. However, this also slightly affects the characteristic of the middle band. The bandwidth of the upper band is at its widest when *W9* is at 2.9 mm. Figure 7 shows the simulation results of the return loss with various *W9* parameter values for *stub2*. The last optimized result is shown as the red curve in Figure 7.

**Figure 8:**
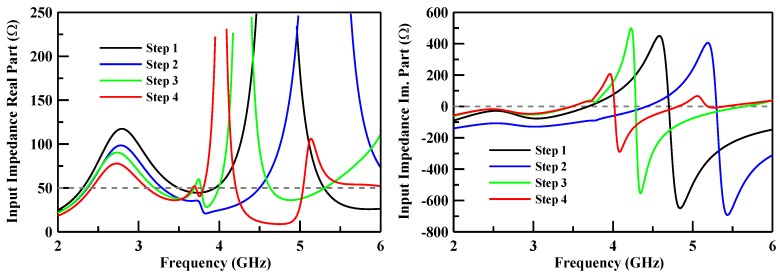
Variations of the impedance using the different models in the optimization processes.

The 10 dB bandwidth of the lower band ranges from 2.30 to 2.70 GHz, the middle band ranges from 3.25 to 3.72 GHz, and the upper band ranges from 5.15 to 5.95 GHz. These simulated results fulfill the spectrum requirements of LTE bands and cover the 5G system (Sub 6 GHz spectrum). Otherwise, these operation bands can cover the LTE-U band in the 5G system. The variation of impedance for each step in the design process is shown in Figure 8. The imaginary part of the impedance is close to zero ohm during the optimized processes.

**Figure 9:**
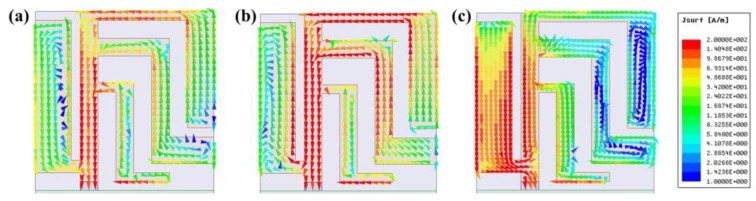
Surface current distribution of the planar USB dongle antenna at (**a**) 2.45 GHz, (**b**) 3.51 GHz, and (**c**) 5.45 GHz.

The surface current distribution of the planar USB dongle antenna is shown in Figure 9. Specifically, Figure 9a,b illustrates the current distribution at 2.45 GHz lower band and 3.51 GHz middle band, respectively. These results are not easy to differentiate, using the characteristics of the lower band and the middle band. The surface current distributions are similar to the results shown in Figure 3 for *arm1*. However, there are conspicuous coupling effect on both stubs, *stub1* and *stub2*. These two stubs possess not only the function of impedance tuning but also the coupling effect. On the other hand, Figure 9c illustrates the current concentration on the main antenna *arm2* at 5.45 GHz upper band. This result shows that the upper band is resonated by *arm2*. Moreover, Figure 9c shows that the design satisfies the resonated condition of a quarter wavelength.

## 3. Experimental Results and Discussion

**Figure 10:**
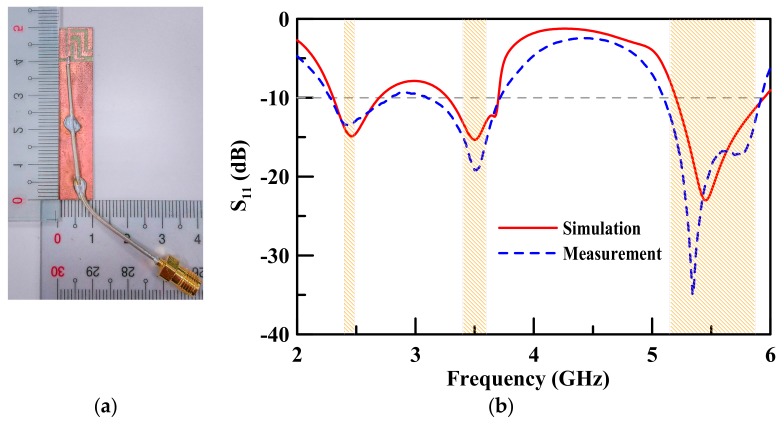
(**a**) Sample of the fabricated antenna; (**b**) comparison of the simulation results and the theoretical measured results in terms of return loss.

Figure 10a shows the planar USB dongle antenna assembly that is fabricated on an FR4 substrate. The proposed antenna was simulated using the EM simulator ANSYS HFSS. Figure 10b shows both the measured *S_11_* of the proposed antenna as well as the simulated results. The measured 10 dB bandwidth of the lower band range is 2.27–2.79 GHz, the middle band range is 3.10–3.72 GHz, and the upper band range is 5.10–5.92 GHz. The measured results were based on a 10 dB return loss threshold that could cover an LTE inclusive of the 2.30–2.69 GHz, 3.40–3.70 GHz, and 5.15–5.85 GHz bands, as well as the entire frequency bands of the 5G system (frequencies lower than 6 GHz). The LTE-U band for the 5G system is included in WLAN, which the proposed antenna can support as well. As for the simulated results, it clearly shows the same trend as that of the theoretical measured results. The three operating bands work at 2.53 GHz, 3.41 GHz, and 5.51 GHz, with a bandwidth of 20.6%, 18.2%, and 14.9%, respectively.

**Figure 11:**
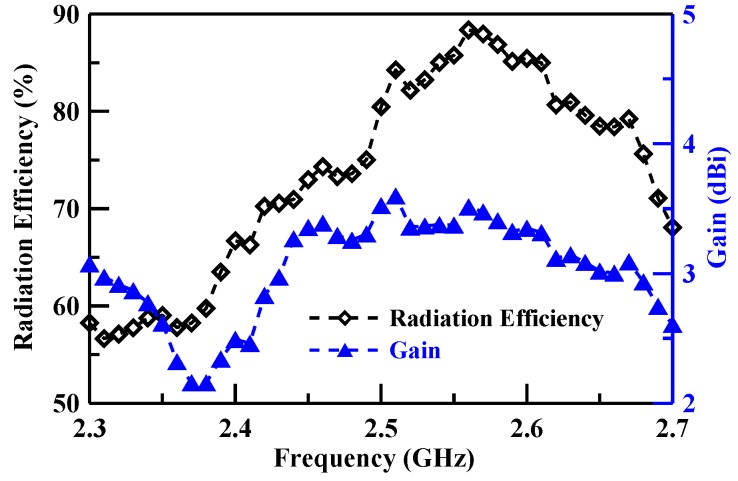
Lower band gain and radiation efficiency measurement results.

**Figure 12:**
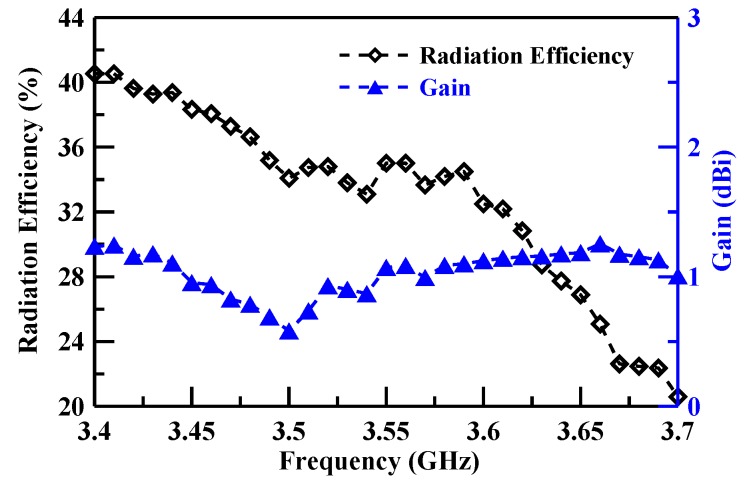
Middle band gain and radiation efficiency measurement results.

**Figure 13:**
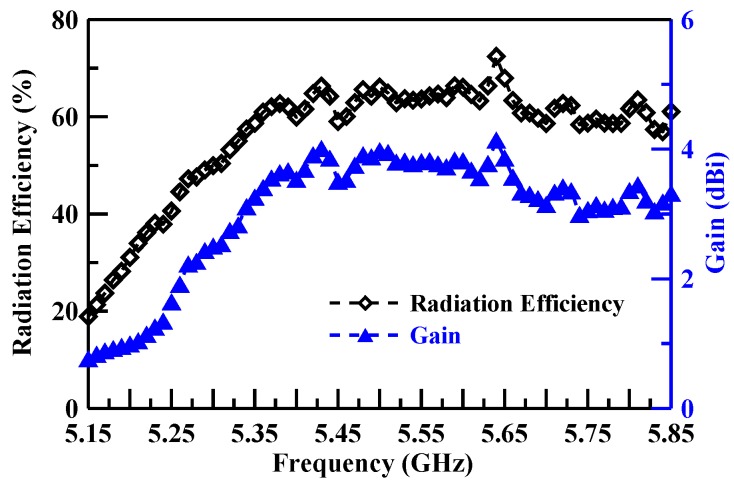
Upper band gain and radiation efficiency measurement results.

Figure 11, Figure 12 and Figure 13 illustrate the peak gain and radiation efficiency. Figure 11 shows a peak gain of 2.16–3.51 dBi with a radiation efficiency of 56.65–88.37% for the lower band. The middle band shows a peak gain of 0.60–1.24 dBi with a radiation efficiency of 20.59–40.54%, as illustrated in Figure 12. The upper band peak gain of 0.84–4.13 dBi with a radiation efficiency of 18.91–72.42% is shown in Figure 13.

**Figure 14:**
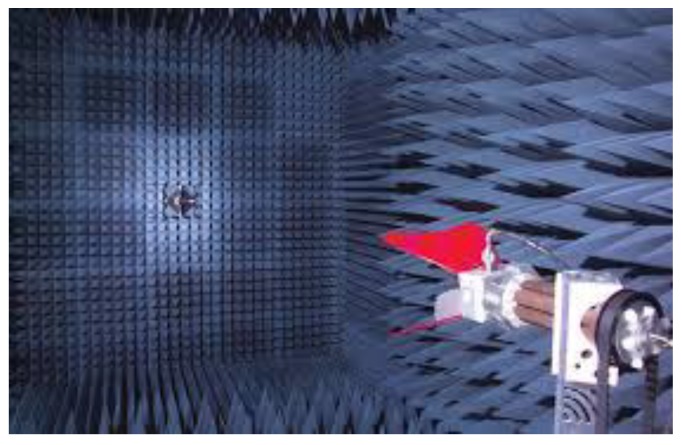
Photograph of the anechoic chamber.

**Figure 15:**
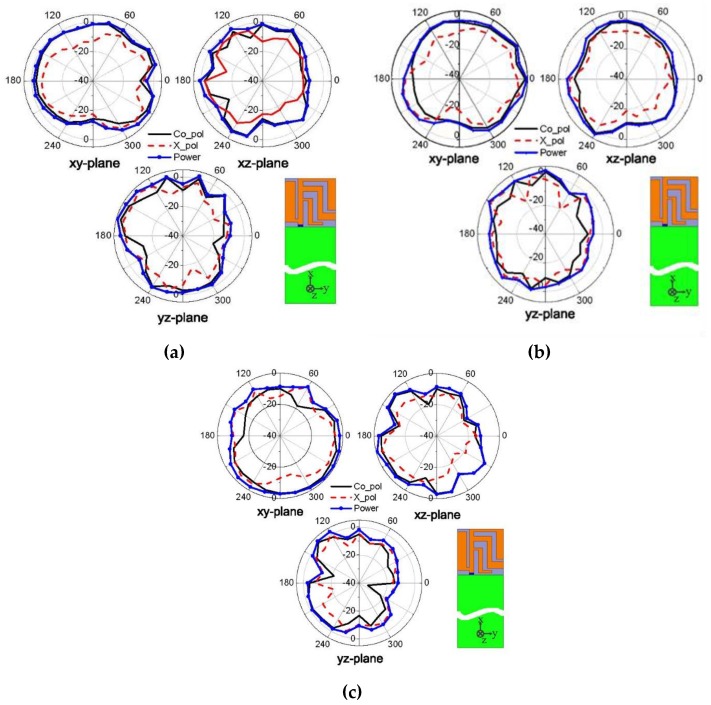
Two-dimensional normalized radiation pattern of the planar USB dongle antenna at (**a**) 2.45 GHz, (**b**) 3.51 GHz, and (**c**) 5.45 GHz.

**Figure 16:**
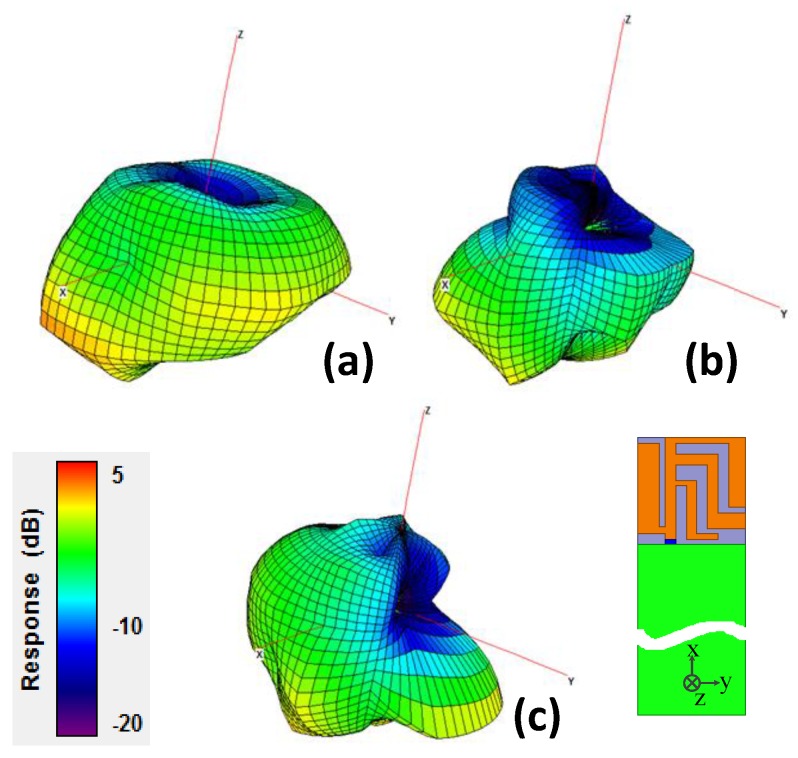
Three-dimensional normalized radiation pattern of the planar USB dongle antenna at (**a**) 2.45 GHz, (**b**) 3.51 GHz, and (**c**) 5.44 GHz.

To further investigate the radiation performance of the proposed antenna, a 3D far-field antenna measurement system was adopted, with its photograph shown in Figure 14. The system is mainly composed of an anechoic chamber, a vector network analyzer, position controller, and a central control computer. The anechoic antenna chamber with a dimension of 7.3 × 3.7 × 3.7 m^3^ has the installed pyramid-shaped absorbers inside the chamber to build a propagation environment without reflection. All of the equipment is connected to the central control computer. By using the position controller, it can rotate and precisely position the antenna at a specific angle by setting the values of the theta and phi angles. The network analyzer Agilent E5071C that measures the frequency with a scope ranging from 100 kHz to 8.5 GHz was used to measure the *S* parameters of the antenna, the gain pattern, and the radiation efficiency of the antenna. The measured radiation patterns are at 2.45 GHz, 3.50 GHz, and 5.40 GHz in the *xy*, *yz*, and *zx* planes and are plotted in Figure 15. The 3D normalized radiation patterns are illustrated in Figure 16. Based on the measured radiation pattern, the proposed planar USB dongle antenna shows similarities with an isotropic antenna.

The operating frequency bands and radiation efficiency of the proposed antenna achieved the target design goals in this study. The area of the proposed antenna is 10 × 10 mm^2^. The peak gain is within the range of 0 to 4 dBi. The radiation efficiency of each frequency band is higher than 10% and satisfies the design goal. Table 2 lists the performance comparison of the proposed antenna with previously proposed antennas in terms of system ground size and antenna size [25,26,27,28,29,30,31,32,33,34,35,36,37,38,39,40,41,42,43]. Three types of planar antennas were compared in Table 2, namely the tridimensional structure, single-sided planar antenna, and double-sided planar antenna. In [27,42], the structure of the antenna is tridimensional. The manufacturing process of a tridimensional antenna becomes more difficult as the size of the antenna is reduced. The planar antenna is preferred for antenna design since there is no complex welding and folding needed in the fabrication process of the planar antenna. There are two kinds of planar antenna, the single-sided design and the double-sided design. The single-sided design is simpler than the double-sided design. A single-sided planar antenna is used to design the proposed antenna in this study with a 10 × 10 mm^2^ measured antenna size. Chung et al. [43] also proposed the same size of antenna, 10 × 10 mm^2^. However, their proposed design had the antenna extended to the back of the substrate, making it a double-sided planar antenna. The proposed design in this study is simpler than the design in [43] with an advantage of having wider supported frequency bands.

Table 3 lists the comparison of the proposed antenna design with previously proposed antennas in terms of their supported frequency bands. The results of Table 3 show the difficulty in designing a multiband antenna within a small area. The properties of the antenna must satisfy the conditions and limits in communication systems. The coverage of the frequency bands is the first condition. The following designs support three frequency bands [28,30,31,33,34,35,36,37,39,40,41,42,43]. The next property to consider is the antenna size, where that in [43] and the proposed design in this study are the smallest, as listed in Table 2. The measurement results of the peak gain that are within the 0–4 dBi range are in [35,39,41] and this study. The peak gain is 5–7 dBi in [43] however its design is not omnidirectional enough. The proposed antenna design in this study satisfies the requirements for both LTE operating bands, 2.30–2.69 GHz, 3.40–3.70 GHz, and 5.15–5.85 GHz, and a 5G system (sub 6 GHz spectrum) operating band, 3.40–3.60 GHz. The LTE-U band of a 5G system is also included in the supported frequency bands of the proposed antenna in this study.

## 4. Conclusions

A high-performance antenna has two main indicators. The first indicator is the function of the antenna, namely its supported frequency bands, gain, and radiation efficiency. The second indicator pertains to the structure of the antenna. The size of antenna needs to be as small as possible and under its physical limited condition. Under Chu’s limits, the radiation efficiency and bandwidth are limited by the size of the antenna. The Friis transmission equation can estimate the radiation efficiency that the system needs under the conditions of the operational environment. The omnidirectional antenna is an ideal mode for the USB dongle antenna; the ideal peak gain must be close to 0 dBi. Following the said conditions enables the construction of an antenna design that works within its expected performance. Moreover, manufacturing of the antenna must be simplified in order to reduce the cost.

A compact multiband planar USB dongle antenna has been designed to cover the LTE, 5G system (Sub 6 GHz spectrum), Bluetooth, and frequency bands for WSNs. The radiation efficiency achieved is 10% for each band. The peak gain for each band is approximately 0–4 dBi. The main antenna has a compact size of 10 × 10 mm^2^ and a volume of 10 × 50 × 0.8 mm^3^. The main advantage of the proposed antenna is that it can support the triple band operation with a small size and simplified process for manufacturing. The proposed antenna can support a 5G LTE system, which can be embedded in a wireless communication device for WSNs applications.

## Figures and Tables

**Table 1:** Dimensions of the planar USB dongle antenna: (**a**) length and (**b**) width.

**(a) Length.**	
**Length**	**(mm)**
*L*	50.0
*L1*	10.0
*L2*	8.5
*L3*	0.7
*L4*	9.5
*L5*	5.5
*L6*	7.0
*L7*	0.5
*L8*	6.5
*L9*	1.5
**(b) Width.**	
**Width**	**(mm)**
*W*	10.0
*W1*	2.0
*W2*	5.0
*W3*	3.0
*W4*	1.0
*W5*	1.5
*W6*	0.5
*W7*	3.5
*W8*	2.5
*W9*	2.9

**Table 2:** Comparison of the proposed antenna with previously proposed antennas in terms of system ground size and antenna size.

**Published Literature**	**System Ground Size** (L (mm) * W (mm)	**Antenna Size** (L (mm) * W (mm)	**Planar Antenna**
Al-Khaldi et al. [25]	33 * 13	7 * 33	Yes (single sided)
Gonçalves et al. [26]	20 * 29	9 * 29	Yes (single sided)
Saini et al. [27]	44 * 25	20 * 18	No
Yang et al. [28]	54 * 15	50 * 33.55	Yes (double sided)
Chen et al. [29]	45 * 13	13 * 10	Yes (single sided)
Ullah et al. [30]	35 * 13	35 * 40	Yes (double sided)
Saxena et al. [31]	30 * 13.5	30 * 21.5	Yes (double sided)
Hsu et al. [32]	50 * 19	50 * 31	Yes (double sided)
Kou et al. [33]	40 * 11	40 * 29	Yes (double sided)
Li et al. [34]	18 * 8.7	18 * 25.3	Yes (double sided)
Pandit et al. [35]	21 * 7.3	21 * 16.7	Yes (single sided)
Swathi et al. [36]	40 * 17.5	40 * 27.5	Yes (double sided)
Tangthong et al. [37]	20.8 * 19.2	25 * 16.1	Yes (single sided)
Yu et al. [38]	35 * 10	35 * 25	Yes (double sided)
Kuma et al. [39]	24 * 5	24 * 25	Yes (single sided)
Kim et al. [40]	40 * 15	40 * 35	Yes (single sided)
Ali et al. [41]	22 * 8.3	22 * 16.7	Yes (double sided)
Wong et al. [42]	150 * 75	150 * 4	No
Chung [43]	35 * 10	10 * 10	Yes (double sided)
This study	40 * 10	10 * 10	Yes (single sided)

**Table 3:** Comparison of the proposed antenna with previously proposed antennas in terms of supported frequency bands.

Published Literature	Lower Band (GHz)	Middle Band (GHz)	Upper Band (GHz)
Al-Khaldi et al. [25]	2.50–3.50	NA	5.00–5.50
Gonçalves et al. [26]	2.30–2.69	NA	NA
Saini et al. [27]	2.30–2.70	3.40–3.60	NA
Yang et al. [28]	1.39–1.48	1.75–4.20	5.04–6.00
Chen et al. [29]	2.40–2.484	NA	4.70–5.825
Ullah et al. [30]	2.30–2.69	3.40–3.70	5.15–5.85
Saxena et al. [31]	2.30–2.62 2.63–2.90	3.30–4.80	5.50–8.02
Hsu et al. [32]	1.43–3.29	NA	NA
Kou et al. [33]	2.21–2.53	3.20–3.83	5.41–8.37
Li et al. [34]	2.41–2.63	3.39–3.70	4.96–6.32
Pandit et al. [35]	2.35–2.53	3.20–4.26	5.24–6.06
Swathi et al. [36]	1.68–2.71	3.26–4.06	5.03–6.25
Tangthong et al. [37]	2.29–2.98	3.23–4.16	5.08–6.38.
Yu et al. [38]	NA	3.20–3.90	5.75–5.85
Kuma et al. [39]	2.50–2.71	3.37–3.63	5.20–5.85
Kim et al. [40]	2.39–2.59	3.10–3.57	5.45–6.50
Ali et al. [41]	2.26–2.57	3.27–3.60	5.69–5.98
Wong et al. [42]	2.41–2.63	NA	5.15–5.85
Chung [43]	2.30–2.69	3.40–3.70	5.15–5.85
This study	2.27–2.79	3.11–3.72	5.10–5.92
